# Exploring Yoga Behaviors among College Students Based on the Multi-Theory Model (MTM) of Health Behavior Change

**DOI:** 10.3390/ijerph20146395

**Published:** 2023-07-19

**Authors:** Chia-Liang Dai, Ching-Chen Chen, Manoj Sharma

**Affiliations:** 1 Department of Teaching and Learning, College of Education, University of Nevada, Las Vegas, NV 89154, USA; 2 Department of Counselor Education, School Psychology, and Human Services, College of Education, University of Nevada, Las Vegas, NV 89154, USA; ching-chen.chen@unlv.edu; 3 Department of Social and Behavioral Health, School of Public Health, & Internal Medicine, University of Nevada, Las Vegas, NV 89154, USA; manoj.sharma@unlv.edu; 4 Department of Internal Medicine, Kirk Kerkorian School of Medicine at UNLV, University of Nevada, Las Vegas, NV 89154, USA

**Keywords:** health benefits, mindfulness meditation, wellness education

## Abstract

During college years, perceived stress is the top reported hindrance to well-being and academic success. Data on the acceptance and perceived benefits of yoga among college students are limited. A purposive sample of college students (n = 79) from a course centered on Vinyasa Yoga and Mindfulness Meditation were recruited. Comprehensive yoga journaling data were collected, and a directed content analysis along the constructs of the multi-theory model (MTM) of health behavior change was utilized. The advantages of yoga that emerged were improved physical performance, reduced perceived stress, acceptance of oneself, better well-being, and improved coping. The identified disadvantages were time commitment, lack of motivation, and other competing interests. Learning through small steps, modifications, and identifying multiple sources of confidence helped build behavioral confidence. Practicing at home or at a yoga studio was a common theme for support in the physical environment. Directing negative emotions into purposes was helpful in maintaining the yoga practice. Sustained commitment to the practice also helped maintain the regular performance of yoga. Finally, social support from family, friends, and instructors was vital for continued practice. The study has important ramifications for the development of survey tools for descriptive studies and designing behavior-change yoga interventions in this target population.

## 1. Introduction

College is a challenging transition from adolescence to early adulthood. Anxiety and stress are named the top hindrances to academic success and personal wellness according to a national assessment in the U.S. [1]. Indeed, college may represent a period in which adolescents experience independence by leaving home without parental supervision, managing finances individually, establishing or ending a close relationship, adjusting to a new cultural context, and facing other stressful situations that create stress. Conley and colleagues found that college students experienced increased depression, decreased self-esteem, decreased active emotional coping, and decreased social support from friends across the first two years in college [2], which highlighted that intervention programs are needed, especially during the transition period.

One study aimed to identify health-related risk factors for well-being among undergraduate college students. The researchers found physical inactivity, sleep problems, and tobacco use to negatively influence college students’ well-being [3]. Cahuas and colleagues examined the relationship between physical activity, sleep behavior, and depression symptoms among college students. The findings of the study showed that physical activity and sleep quality predicted depression levels [4]. Similar results were found in a previous study conducted by Ghrouz and colleagues [5]. Physical activity is a protective factor to prevent mental illness and adverse health conditions such as being overweight, obesity, etc., which can, in turn, enhance overall well-being among college students. Yoga, one form of physical exercise and mind–body intervention, along with meditation and relaxation techniques, has gained widespread popularity among a broad population. Yoga-based practices have also been intervened successfully in treating physical and mental symptoms related to anxiety, depression, and stress, and can improve general well-being [6,7]. Yoga is becoming very popular on college campuses where it is being taught for credit as well as for non-credit. An evaluation of a six-week yoga intervention on college students reported a reduction in stress and anxiety levels [8]. Another six-week intervention with college students using Hatha yoga demonstrated a significant reduction in stress and anxiety [9]. More research is needed to examine the impact of yoga courses on college students’ physical and psychological well-being.

The multi-theory model (MTM) of health behavior change attempts to explain changes in a specific behavior through two components: initiation and sustenance [10]. There are three constructs that influence the initiation of health behavior change; they are participatory dialogue, behavioral confidence, and changes in the physical environment. For the sustenance of health behavior change, the three constructs are emotional transformation, practice for change, and changes in the social environment [9]. The constructs of initiation and sustenance models within the MTM framework have received support for their ability to explain various health-related behaviors. In particular, researchers have applied the MTM theory to assess behaviors related to physical activity [11,12,13,14,15,16,17]. Hayes and colleagues conducted a cross-sectional study utilizing the MTM of health behavior change theory to investigate changes in yoga practice among college students and found that changes in the physical environment and behavioral confidence explained the initiation of yoga practice while practice for change predicted the maintenance of yoga practice behavior. The study suggested that the MTM of health behavior change provides a useful structure in explaining the intent to start and maintain yoga behavior among college students [18]. While prior studies regarding MTM and physical activity behaviors are growing, yoga practice was rarely studied. To fill the gap, the current study employed a qualitative approach utilizing the MTM as a guide to explore participants’ yoga practice experience and their perceived influence on health.

Data regarding exercise-related courses’ acceptance and perceived benefits on health maintenance among college students is limited. The purpose of the study was to inquire about yoga practice behaviors and the perceived impact of yoga practice on physical and psychosocial well-being among college students by utilizing the framework of MTM health behavior change.

## 2. Materials and Methods

### 2.1. Participants

A purposive sample of college students from a health and fitness course, centered on Vinyasa Yoga and Mindfulness Meditation, offered at a southwestern university were recruited. Comprehensive yoga journaling was requested from all students. In total, 79 (72 females and 7 males) out of 141 (56%) students submitted their yoga journal. The sample allowed for data saturation. No control group was used.

### 2.2. Course Description

The 15-week, one-credit course was titled Yoga for Health and Fitness. The class was designed to promote wellness and fitness through the practice of yoga postures and breathing skills as well as with a concentration on mindfulness meditation. Students were taught yoga postures, breathing exercises, meditation, and relaxation in a 50-min session. The class met twice weekly for 15 weeks for a total of 30 sessions. Each class session typically consisted of guided breathing exercises and mindfulness meditation (5 min), yoga posture practices (35 min), and guided relaxation and wrap-up discussion (10 min). The same yoga components and sequence of yoga postures were used in each class. In the yoga posture practices, alternative postures and body movements were provided to meet participants with different needs. The instructor was hired through the Recreation and Wellness Center at a Research 1 university. The instructor was not a registered yoga teacher, yet the instructor had instructed other yoga sessions at the university and provided yoga classes in other fitness studios for years. 

### 2.3. Procedure

The Institutional Review Board approval (Protocol #: 1418598-1) was obtained from the University of Nevada, Las Vegas. Students were asked to submit the yoga journals by answering the following open-ended question: describe your experiences of yoga practice in this class. Students were informed that the submission of the yoga journals was voluntary and that the submission or not would not affect their grades. There was no specific requirement regarding the length or format of the paper submission. Students turned in their yoga journals to the WebCampus Learning Management Platform used by the university. A doctoral-level graduate assistant who had completed the qualitative research methodologies class and served as a research assistant in other research projects handled the raw data. Specifically, the graduate assistant downloaded the ZIP file which archived yoga journals submitted by the participants. The graduate assistant unzipped the file and compiled the yoga journals into a Word file. In the Word file, no participant’s name appeared; instead, a coded number was used to represent each participant. For example, in the coded number 10621-1M, 10621 indicated the class session, 1 indicated the order of the yoga journal in the ZIP file, and M indicated that the participant was a male.

### 2.4. Data Analysis

Directed content analysis [19] along the seven constructs of the multi-theory model (MTM) of health behavior change [10] was utilized on the collected data. The constructs for initiation of yoga behavior in MTM are advantages, disadvantages, behavioral confidence, and changes in the physical environment, while the constructs in the sustenance model are emotional transformation, practice for change, and changes in the social environment. Two researchers completed the initial coding independently. The initial coding process was carried out by two researchers who were not part of the class, thus maintaining anonymity. The results of the initial coding were discussed and reconciled among the three researchers. The first coder’s research centered on using lifestyle-based behavior approaches, especially yoga, to promote personal health and wellness. The second coder conducted intervention research targeting mental health and wellness issues. The third coder was an expert in the theory of health behavior change as well as complementary and alternative medicine. Three coders have been trained in conducting qualitative research, have published qualitative studies, and were familiar with the constructs of MTM of health behavior change. An example of content analysis aligning the constructs of the MTM behavior change can be found in Table 1.

## 3. Results

The study aimed to explore participants’ perceptions of yoga practice in a class. At the end of this 15-week course, 79 (72 females and 7 males) out of 141 students submitted their yoga journal reflecting on their experiences of yoga practice in this course. On average, students attended yoga sessions ranging from 28 to 30 sessions. The constructs of MTM were guided to analyze yoga journal data about participants’ perceptions of participating in the yoga course, and the details are presented in the following section:

### 3.1. Model of Behavior Change-Initiation

#### 3.1.1. Advantages

The themes that emerged as advantages were improved physical performance and condition, reduced perceived stress and anxiety, acceptance of themselves and increased mindfulness, feeling recharged and refocused, improved coping, and better overall well-being.

##### Improved Physical Performance, Health Condition, and Exercise Habits

Participants mentioned changes in body function that included improved flexibility, better balance, and increased strength and endurance. Participants often described the changes by measuring their physical performance on a specific yoga posture. For example, one participant described “I can touch my toes” (10621-5F), and many participants noted that they were not able to do it before. One of them noted, “I was gaining a lot of strength in my core. Moving from downward dog to a plank is where I could feel my strength building in my core” (10623-3F). Another participant stated that “I have never been able to have my heels flat on the ground in down dog and I was actually able to do that this semester” (10622-3F).

The yoga practice that improved participants’ health conditions was also discussed. One participant indicated that “I would say my back feels much better. The stretching helps work out the knots that I have in my back” (10621-3F). Notably, participants mentioned that yoga practice helped their injury recovery after surgery. One participant noted, “I had to have surgery on my wrist and experienced some complications after the surgery…poses like a down dog actually helped to stretch the wrist and it felt good” (10622-4F). Another one had back surgery a few years ago and was dealing with the pain that comes with that from everyday living, addressing that “I’ve noticed that my pain levels decreased throughout the semester. I think that the core work we did and the back strengthening exercises really benefited my back” (10621-2F).

Participants also noted that the yoga stretching they practiced in the class benefited their other workouts or sports activities. One described “I have always had problems with my hips when I would participate in sports or workout but yoga help stretch out my hip so I do not have as many problems with my hips” (10623-3F). One participant reported that due to the flexibility improvement, “I noticed that exercises such as squats and deadlifts became easier since my range of motion had increased” (10622-4M). Participants also wrote that they felt increased motivation to exercise daily due to taking the yoga class. For example, one participant noted that “Yoga made me want to start working out every day again to get back on the right track to being in shape” (10623-4F).

##### Reduced Perceived Stress/Anxiety and Recharged

Perceived pressure on academic requirements was the top stressor reported by participants. Besides demanding schoolwork, other stressors that were mentioned included transferring schools, switching majors, experiencing surgery, being away from home, friendships, personal life, jobs, extra-curricular activities, and so many important decisions and events coming up that have caused constant perceived stress. For example, one expressed that “as a pre-medical student, I am always under the stress of deadlines, challenging curriculums, and many extra-curricular involvements” (10623-9F). Participants shared that the yoga practice in the class helped them relieve their stress from other demands and get through the “stressful semester” (10622-2F). For example, one wrote “as a musician, I found that it (yoga) is especially helpful to conquer stage fright and performance anxiety” (10621-8F). Another stated “as a dancer and designer, I definitely need time to devote to myself to reenergize and revitalize my overworked body” (10621-1M). One participant described the yoga practice experience as “allowing time for your body and soul to be grounded back to the earth” (10621-4F).

Participants discussed yoga practice not only helped them feel less stress/anxiety but also helped them feel recharged and refocused on the tasks in their life. Common expressions participants noted were that “after Yoga I always feel refreshed and ready to start my day” (10627-3F). One participant shared that “surprisingly enough yoga actually woke me up, even though it was very calming and relaxing I would always feel wide awake and focused for the day” (10621-10F). Another participant reported that “I had a 9 am class right after yoga and I felt so much more focused in there on those days than I did any other day that I did not have yoga” (10621-10F).

Some yoga postures such as the Child pose and Shavasana pose were commonly referred to by participants as their favorite poses for the mind and body to be relaxed. Along with yoga postures, meditation and breathing practiced in the class were also reported as helpful in relaxing the mind. One described,

“*While it (yoga) is a workout, the meditations that we did and the child’s pose really helped me relax. I especially enjoyed the long break we had at the end. It helped end with class in a really relaxing way and helped me recharge and go on with the rest of my day*”

(10621-3F)

Participants also often credited their stress management and productivity to yoga practice. For example, one wrote, “taking yoga this semester has helped me to set me aside for myself to relax, stretch, and reflect” (10623-10F). Another expressed that “I had resigned myself to the fact that I would always deal with my anxiety, that is, until I took this class” (10624-2F). Likewise, one reflected that “the hour (yoga class) I took out of my day a few times a week really helped me focus on the issues at hand and deal with them in the best possible way I could” (10623-6F). Lastly, one recalled that “On days that I had a big exam I looked forward to yoga because I needed to clear my mind and focus on my body” (10621-8F).

##### Acceptance of Oneself

Participants claimed yoga practice helped them accept themselves and their current status both in body and mind. For example, one noted that “If you really focus during yoga practice you really do find inner peace within yourself. Personally, I love finding inner peace and being happy with myself. I think that is important in life as well as being happy with who you are and not worrying about what you are not” (10621-11F).

Quotes regarding increased awareness of body condition were frequently observed. One noted that “I think that one really important thing I learned from this class is how important it is to listen to your body and what it’s telling you” (10621-2F). Another shared that “Yoga has also helped me to appreciate my body. I have struggled with negative body image” (10621-6F). Another wrote that “I don’t care if I lose my balance or have to go into child’s pose because the downward dog is just killing my shoulders, I don’t care. This class is a judge-free zone and I really admire that” (10622-5F). Another discussed,

“*I think one of the best things about yoga, is there is no pressure to be as good as anyone else. You are supposed to do what your body tells you and not push it. It is nice to find a physical activity where you don’t feel ashamed for not being able to fully do something or do it 100% correctly*”

(10622-2F)

##### Increased Mindfulness

Participants also mentioned that yoga practice helped them to be better at being mindful. One journaled that “Normally when we lie down and meditate at the end I find myself coming to peace. I just forget everything going on in my life and it really is nice to feel that way” (10621-11F). Another reported that “Yoga allowed me to stop from my stressful everyday life, and just take a moment to unwind, breathe, and focus on myself” (10622-7F). One participant described how she acknowledged her own emotion and discussed her responses.

“*I do not handle stress very well and I tend to get angry with myself as well as others for my own problems. I don’t have a lot of self-confidence and I worry a lot about what other people think of me and I shy away from situations where I have to talk to new people. During yoga practice, I started to really focus on connecting with my breath and just being in the moment … Instead of dwelling on negative emotions for long periods of time, I allow myself to feel whatever it is I am feeling and I am grateful that I am able to do so and then I let it go*”

(10623-7F)

Participants also credited yoga practice for better concentration and indicated that yoga practice allowed them to set time for themselves to engage in self-care. One addressed that “When I am having a stressful week it is nice to know that I always have my two hours of yoga a week to be able to relax and forget about all of my stress” (10627-7F). Other participants reflected that “I think this yoga class has really helped me this semester stay focused and to only remember the important things in life and to make time for myself” (10622-5F); “The class always had a calming and relaxing feel to it where I could breathe and take a moment for myself” (10621-2F).

##### Improved Coping

Participants commented on the positive impact that yoga practice has on their coping mechanisms to manage their stress. One mentioned that “now when I find myself feeling anxious, if it is possible, I try to meditate or work on some of the poses that we learned in class … my worries are less significant and I find that I rarely have panic attacks” (10624-2F). One participant noted,

“*Throughout the semester when I had large assignments coming up, midterm week, and even weeks where I just didn’t have enough time in the day I would stop for about 15 min and focus on my breathing and practice a few moves*”

(10622-3F)

Participants journaled that they performed breathing and stretching techniques learned in the class when they felt tense in daily life. One described “I also really enjoyed the meditation and breathing aspect. I am very easily stressed out and it was great for me to learn different outlets to relieve that stress” (10621-1F). “Before every exam this semester I would take half an hour and meditate” (10628-11F). Another participant wrote that “I now practice yoga meditation at night before I go to sleep and it helps me relax after a stressful day” (10623-5F).

The breathing techniques and meditation practiced during yoga served as a coping mechanism during tough life events among participants. One wrote that “The breathing circle has helped me calm myself down when I need to. As you know, my friend recently passed away. This was very hard to accept and deal with. Yoga has helped me with this process (grief)” (10621-3F). Another noted that “I was very thankful for learning different ways of meditation that helped me mentally get through my injury and illnesses” (10623-9F).

Participants often mentioned they were able to apply the skills learned in class to improve their mood in daily life. One reflected,

“*For me a yoga class is much more than an exercise, it’s about relaxing and being able to control your breathing. I really appreciated how much time we took to meditate, that was a very positive aspect of the class for me. The 4-2-6 breathing technique is something I have used on almost a daily basis since learning it*”

(10622-3F)

Participants expressed the benefits of connecting breath to the body and mind. One described it as follows: “I think that when I do practice yoga it helps me breathe through my feelings and find a right state of mind” (10623-6F). Another noted that “I have noticed that practicing yoga has made me a happier person and helped me deal with anxiety and stress. I am better able to focus on the positive side of things than allowing negative situations to influence me” (10623-9F).

##### Better Overall Well-Being

Most of the participants described that yoga practice is rewarding and has been helpful in bettering their well-being physically and psychologically. It is “healthy for the mind, body, and spirit” (10628-12F). One claimed that “my body has physically felt a hundred times stronger which has played into my mentality” (10627-1F). One wrote, “Since taking this class I find myself more confident and calmer. I am proud of what my body can do and how flexible I have become” (10624-2F). Another reflected that “I found that yoga really helped my health and well-being as well. I felt much more relaxed and stretched out physically, while also felt my flexibility returning and some of my strength building up again” (10622-2F).

Participants also reported that yoga practice contributes to health-enhancing behaviors which benefit overall well-being. One reported that “When I finished my day with yoga I slept much better because my mind was well rested as well as my body” (10628-6F). One noted,

“*Yoga also causes me to make healthy choices. After yoga class, I feel energized and ready for the rest of the day. I eat better on the days I practice yoga and am more energetic. Yoga also helps me stretch out before I go to the gym. Each morning I practice Yoga my body is less achy and feels more limber*”

(10623-5F)

Participants expressed that the benefits of yoga practice include a variety of dimensions of well-being. One journaled, “NO, stretching is not the ONLY thing we do. Yoga has really surprised me. I believe I have become a strong, more focused, positive person after being in this class” (10622-5F). One addressed that “Yoga has helped me academically, socially, mentally, and physically. It is something that I want to continue doing and improve upon because I am so happy about the positive changes that it has brought to my life” (10621-12F).

#### 3.1.2. Disadvantages

Some barriers to yoga practice that the participants noted included time commitment, lack of motivation, confidence, and other competing interests.

##### Time Commitment

Participants addressed that the yoga class offered in the early morning made it hard for their attendance. “The only struggle with yoga for me was waking up early for it. I normally love doing yoga but the early 8 am seemed like a struggle” (10621-11F). Another participant wrote,

“*I can say that some days were very hard to get up and get engaged in the class. There will come days when you often feel exhausted, and just want to curl up and not get out of bed for a couple of hours. So, the thought of getting up and going to yoga was quite draining at times*”

(10621-5M)

##### Lack of Technique/Skills

Participants discussed the lack of technique/skills when they experienced the new physical exercise-yoga. One reflected that “the first couple of classes were not very easy for me; I wasn’t able to control my breathing which made it difficult to relax” (10621-1F).

“*It was hard for me to stay focused even during meditation. I couldn’t clear my head and forget my thoughts; they still ran through my mind keeping me from truly being at peace. I also found some of (well most of) the positions very uncomfortable*”

(10621-7F)

Participants also indicated that practicing yoga outside of class was challenging. One participant addressed that “I find it is a lot harder to do yoga outside of class without an instructor. It is hard to find a calm place to practice yoga other than the classroom” (10622-10F). Another noted that “I have discovered that it is much harder to practice yoga at home. I think I have too many distractions” (10628-12F).

##### Physical Condition

Another disadvantage of yoga practice participants expressed was the concern about their physical condition. For example, participants felt that they were not flexible to perform yoga. One noted that “my hips and hamstrings were extremely inflexible and tight. I struggled to do basic positions and I was worried that this class was not for me” (10622-4M). Another participant claimed that “my least favorite pose was the downward dog, I don’t know why I despise that position but it was extremely uncomfortable for me” (10628-1F).

Some participants had limited body performance due to previous surgeries on a body part that were mentioned as a disadvantage. One participant wrote that “I am disappointed because I am limited when doing some moves due to injuries that I have previously received” (10623-2F).

##### Other Competing Interests

Participants mentioned that competing interests and commitments prohibited them from participating in yoga classes, mostly referred to as projects/requirements required by other classes or duties. One mentioned that “I was quite upset when my classes and theater work/show schedule kept me from attending yoga class” (10622-2F). Another shared that “there were a couple of classes I missed for a field trip to Chicago or if I had pulled an all-nighter and was still trying to complete my project before its due date, I was not able to make it to class” (10621-2F).

#### 3.1.3. Behavioral Confidence

##### Learning through Small Steps, Modifications, and Identifying Multifarious Sources of Confidence

Participants commented on the impact repetition of practice has on the mastery of yoga posture/movement. One wrote that “at first some of the poses and positions were difficult to achieve but with practice I found them becoming easier and easier over time” (10621-8F). Similarly, another answered, “I really liked the pace of the class and how we would do the same poses and gradually as the semester went on add new ones” (10621-10F). One also reflected that “I used to hate going into the “down dog” pose because it was so hard for me to keep my balance and hold myself up for so long” (10621-6F), but by practicing throughout the semester, the participant had become stronger and appreciated the movements of yoga.

Participants endorsed that the modification of yoga posture and the choice they were given in the yoga practice allowed them to master it at their own pace. One participant noted that “Yoga is a personal exercise. Each pose can be altered to fit an individual’s skill level and it is not a competition. I do not have to feel inferior for not being able to do something” (10621-6F). Another responded, “although sometimes the yoga poses were quite challenging, a simpler version of that pose was always an option, which I think was great” (10622-2F).

Participants noted that the awareness of their body’s capability impacted their perceived competence. One participant discussed that “every time I do a different pose, I learn more about what my body is capable of” (10621-6F). Another expressed, “I loved how we got to go at our own pace, and each week we would work our way up to the harder poses” (10621-4F). One wrote that one of her favorite poses was the warrior pose, “it draws from your core strength and forces you to push yourself just a little bit further” (10621-2F). Participants also discussed that accepting the challenge has allowed them to see the growth of their competence. One journaled that “I was challenged by the class in a good way and found myself trying to push to see how far I could go with it” (10621-3F). Another claimed that “I find that yoga progressively gets more difficult as the class goes on. I really like the challenges yoga provides me and like to keep practicing until I get it right” (10623-5F).

Overall, participants reflected that the repetitions and alternatives of a yoga posture kept them unharmed and motivated to perform yoga. One wrote,

“I loved the use of different poses and the option of pushing your body farther because it really helped me better myself and my body… The use of repetitiveness was nice because we always returned to downward dog and the resting pause was a much needed break after a few stretches” (10622-1F).

#### 3.1.4. Changes in the Physical Environment

##### Learning at Home or at a Yoga Studio

Participants stated that they started to learn yoga at home. One wrote, “after this class, I do yoga to stretch and whenever I am stressed because it is very good for relaxing” (10623-3F). Another participant indicated that “Yoga is now a part of my daily routine before I run” (10627-6F). Besides yoga postures, participants also learned breath practice at home. One noted that “the breath aspect that we have worked on, I have also taken very seriously outside of the classroom and practice. I use it at home to relax and calm myself down if I am stressed or worried” (10621-12F). Some participants also mentioned how they prepared themselves for learning yoga at home. For example, one journaled that “having a yoga mat at home is also more motivation since it sits in right by my door and I pass it every day” (10621-7F).

The atmosphere created in the yoga class was noted by participants as a factor that enhanced the learning experience. One addressed that “I definitely like having an instructor and being in class better than practicing at home. I don’t quite get the same feeling I do at home as when I’m in class” (10621-2F). Therefore, some participants chose to attend yoga classes at a yoga studio to support their practice. One shared, “I have actually enrolled in a yoga class that meets twice a week at my gym because this class showed me how important flexibility is in everyday activities” (10622-4M).

### 3.2. Model of Behavior Change-Sustenance

#### 3.2.1. Emotional Transformation

##### Directing Negative Emotions to Purposes

Prior to the yoga class, several participants were skeptical and had stereotypes that yoga is for a certain type of population. For example, some participants thought yoga is lame, just for fun, looks awkward, and underestimated the effects of yoga mentally and physically. One noted that “in the beginning, I took yoga to just chill out my last semester in undergrad but quickly realized it was more than a filler class” (10623-4F). After the yoga practice during the semester, participants reflected that they gained insights into yoga and perceived the benefits of regular yoga practice. “Throughout the semester I was able to figure out what my favorite aspects of yoga were and how they influenced my day. I loved stretching all of my limbs and doing difficult poses that challenged my body” (10621-1F). “The one main thing that yoga helped me realize is that the stress will still be there no matter how much I think about it and that it’s always better to do yoga when you are stressed because you need to give yourself time to be less tense” (10621-4F).

Although yoga sessions held in the early morning were a barrier that participants addressed, participants reflected on the positive impact of joining the early yoga practice. One responded “Having to wake up so early was definitely a struggle for me because I am not a morning person, but it got easier and easier. It was such a great way to wake up and get my day started” (10621-12F). Another indicated that “Although yoga started at 8 am and I happen to not be an early riser, I could actually look forward to it. It was a great way for me to start the day, after a good yoga class I could enjoy what the rest of the day had in store for me” (10621-5M). Another journaled,

“*Yoga has also made me realize that getting up and starting your day is something healthy that should be practiced. Yoga helped me go to sleep earlier and for the most part get a decent sleep. It helped me manage my time and become slightly more responsible*”

(10621-5M)

#### 3.2.2. Practice for Change

##### Sustained Commitment to the Practice

Participants reported increased behavior and intention to practice yoga at home. “Since yoga practice has ended, I intend to take all aspects of the yoga I have learned and implement them in my daily life … I plan to set aside time each morning before my busy schedule begins to practice yoga” (10623-9F). “I am considering taking yoga classes on a daily bases now or to even do a few sessions at home from time to time” (10621-8F). “Over the break, I plan to practice some poses I have learned this semester and also maybe teach myself some new ones as well. I know I will do yoga consistently throughout my life because of this course” (10621-10F). Participants also wrote about locating relevant resources—for example, yoga DVDs—to support their yoga practice. One mentioned, “I hope to take a weekly yoga class to continue my study in it, however, for now, I will practice on my own and maybe get some yoga DVDs to use as guidance” (10622-2F). One noted,

“*It is important for me to continue practicing yoga for my physical and mental health. I hope to continue to meditate and decrease my stress. I also hope to continue to increase my strength, especially in my arms. I am hoping to find a yoga video to use at home*”

(10622-10F)

Participants also mentioned their intention to continue learning yoga in other settings—for example, taking a yoga class next semester or joining a yoga class in the community studio. One responded that “Taking this yoga class was a true eye opener and I am thrilled to know that I can take my experiences and use them in other yoga classes around here in the future” (10621-5M). One wrote, “I plan to take more health fitness courses because of how much I enjoyed this one. I also plan to attend a recreational yoga course to learn more poses and perfect the ones I learned this semester” (10621-10F). Another reported that “I will continue to practice yoga a couple of days a week because it made me feel better. I know they have sessions at the rec center, and there is also a hot yoga place close to my apartment” (10621-7F).

Some participants have already enrolled and practiced in other yoga classes to continue their journey in yoga. For instance, one participant reported that “I had developed a strong liking for yoga and felt comfortable taking classes outside of this one. I had begun to attend hot yoga at (studio) and LOVE IT!” (10622-1F). “I look forward to further my yoga practice. I would like to learn more moves and even try hot yoga. I think this is something I will continue to practice for a very long time” (10621-3F). One participant concluded that “I will probably continue my yoga practice both at home and with a class” (10621-6F).

#### 3.2.3. Changes in the Social Environment

##### Social Support from Family, Friends, and Yoga Instructors

Perceived support from parents and their involvement in yoga practice helped strengthen participants’ sustenance in yoga practice. For instance, a participant noted that “I didn’t realize how much I enjoyed the class until I started practicing at home and trying to get my family to start practicing with me” (10621-4F). One noted, “I even got my mom interested in practicing every day. It helps her feel less stiff and sore in the morning. Hopefully, if she goes to classes with me I will be more likely to go too” (10623-5F). Another addressed that “My parents were so excited when I told them about this (back pain relief) because they know how bad my back could hurt at times. My dad has even started taking yoga classes to help his back. He loves it so far” (10621-2F).

Participants also noted that the perceived support from the friends and instructor in the yoga class fostered the yoga experience. One described that “Taking yoga this semester has also been a great way to connect with my friends. I enjoy going to class with my friend and socializing” (10621-6F). Another mentioned “I want to thank you the great instructor who is clearly passionate about Yoga and for helping someone like me find a little peace in the morning to start their day” (10621-7F)

“*I also enjoyed how for the most part, the majority of the class seemed committed and eager to learn and try new poses. Surrounding yourself with like-minded individuals and goal intentions is important*”

(10621-13F)

## 4. Discussion

To our knowledge, this is the first qualitative study examining the themes of yoga behavior change among college students based on a behavioral paradigm of MTM. The findings of this study indicate that enrolling in a yoga course may help students establish healthy lifestyles and effective coping mechanisms that can manage stress and enhance overall well-being. This study has important ramifications for the development of survey tools for descriptive studies and designing behavior change yoga interventions in this target population. This study is different from a previous cross-sectional study [18] in using an in-depth, rich, contextual qualitative paradigm that examines the issue from the perspective of the students as opposed to the researchers.

The study found that the advantages of yoga practice include improved physical performance and condition, improved exercise habits, reduced perceived stress and anxiety, acceptance of oneself and increased mindfulness, feeling recharged and refocused, improved coping, and better overall well-being. These perceived benefits of yoga practice reported by participants reveal their improved ability to manage stress. This is also supported by other studies evaluating the effect of yoga on reducing stress and anxiety [8,9,20]. Nevertheless, we found some disadvantages to yoga practice, including time commitment, lack of technique/skills, lack of confidence, physical condition, and other competing interests. Though we found that the participatory dialogue construct is crucial in initiating yoga behavior, a cross-sectional study found that the participatory dialogue was not significant in initiating yoga practice [18]. The findings of our study may imply that physically practicing yoga in class has helped participants perceive the benefits of yoga. Such opportunities for the practice of yoga by college students can influence their decision-making and sway the pendulum toward the perception of greater advantages.

Behavioral confidence is another emerging theme in initiating yoga practice. Our findings align with previous MTM-based studies indicating that behavioral confidence is a strong indicator in initiating physical activity behavior [11,15,16,18]. Findings of this study suggest the constructs can be built by learning through small steps, repetitions, and modifications of posture. Yoga practice can also be initiated by identifying multiple sources of confidence such as assessing one’s competence, recognizing one’s growth, exploring favorite poses, and accepting and preparing for the challenge. However, there is a caveat in this finding as the students took this course for credit and paid the fee which may have influenced this construct and can be considered as a limitation of our study.

We found that changes in the physical environment explain the starting of yoga practice. For this construct, continuing to practice at home and outside class, practicing in a group in a studio/gym, and obtaining a yoga mat can all help foster yoga practice behavior change in real life. Our findings support that the obtainability, availability, accessibility, convenience, and readiness of resources can initiate behavior change. Indeed, exercise performed in a group context has been popular, and the atmosphere created in the group class is attributed to helping motivate attendance [21] and engagement in exercising [22,23]. In the model of the sustenance of health behavior change, emotional transformation, practice for change, and changes in the social environment were statistically significant predictors of other health behavior change among college students [24,25]. In terms of the sustenance of yoga behavior change, for the construct of emotional transformation, we found that it is important to direct negative emotions and perceptions of yoga to meaningful purposes and positive reactions. For the practice for changes, we found that sustained commitment to constant practice in various settings is key to fostering the maintenance of yoga behavior change. In the construct of changes in the social environment, we found that perceived social support from family, friends, and yoga instructors is vital. This can include sharing perceived benefits of yoga practice with family and friends and engaging family or friends into practicing yoga together. In order for students to continue practicing yoga in real life, they must maintain peer associations as those can be beneficial in sustaining the practice. Buddy groups can be formed beyond the end of the semester for long-term engagement. These should be important aspects to consider when designing yoga interventions among college students based on MTM.

### 4.1. Implications for Practice

The use of the MTM of health behavior change is useful for explaining yoga behaviors and practices among college students. This study has important implications for designing interventions and health promotion strategies for yoga behavior change in this target population. Future interventions should engage students in performing yoga practice to perceive the benefits of yoga, modify yoga postures, and provide proper instructions to establish confidence and increase the availability of yoga-related resources such as yoga mats, videos, or yoga classes to help start the yoga practice. In order to sustain the yoga practice behavior, efforts should be made to overcome barriers via directing negative perceptions to purposes, committing to regular practice, and receiving social support from significant others. Yoga courses can be offered for credit at campuses as well as for non-credit. Such an approach would be especially beneficial for beginners. In addition, students should be encouraged to join yoga classes in gyms and studios. Home practice must also be encouraged. At the policy level, colleges and universities should offer and require students to enroll in yoga and meditation classes.

### 4.2. Strengths and Limitations of the Study

To our knowledge, this is the first qualitative study exploring yoga practice behaviors among college students based on the MTM of health behavior changes. The study provided evidence that yoga behavior change can be explained by utilizing the constructs of MTM health behavior change. Several limitations were observed in the study. First, the participants in the study were predominantly female (91%). This limits our understanding of yoga practice behavior for all genders. Additionally, data from 56% of the class were collected. While the aim of qualitative research is not generalizability, and we did obtain data saturation, the results do point to limited transferability. Demographic data of the participants such as age and race/ethnicity were not collected which is another limitation of the current study. Efforts may be made to collect specific demographic information in future studies which may help tailor services or interventions to meet the needs of different groups. The attendance policy was addressed in the syllabus which indicated that two absences may be allowed without penalty (i.e., affect the course grade). However, the exact number of attendance for each student is not available. In interpreting data, care was taken to ensure trustworthiness (close adherence to the protocol), dependability (detailed description of the procedures), confirmability (use of MTM as the framework), and coherence (systematic documentation). However, data triangulation was not applied in this study as it used only one method which is a limitation. Finally, the students were enrolled in a course for credit and thus the results are limited in generalizability.

## 5. Conclusions

To successfully manage stressors that lead to health-related risk behaviors in college life, healthy coping strategies to enhance well-being should be promoted on the college campus. The findings of the study evidenced that one approach is through meditative movement or fitness-related courses, such as yoga, which should lead to overall health-enhancing benefits among college students. Based on the findings, several strategies can be applied at the individual level to promote the start and maintenance of yoga practice behavior.

## Figures and Tables

**Table 1:** Examples of content analysis aligning the constructs of the Multi-theory Model (MTM) of behavior change.

Model of Behavior Change	Constructs of MTM	Quotes from Journaling Data
Initiation	Advantages	“I also really enjoyed the meditation and breathing aspect. I am very easily stressed out and it was great for me to learn different outlets to relieve that stress (reduced perceived stress and improved coping).” “Yoga was a nice balance for my muscles and the rest of my body because it allowed me to stretch and work on strengthening my inner core (improved physical performance).”
	Disadvantages	“Some days were very hard to get up and get engaged in the class. There were some days where you often feel exhausted, and just want to curl up and not get out of bed (time commitment, lack of motivation).”
	Behavioral Confidence	“The different positions are fascinating to me. I think it is great that there are alternative positions in order to give people an option of a way (learning through modification) to allow their bodies to leave yoga class unharmed. I think one of the best things about yoga, is there is no pressure to be as good as anyone else.”
	Changes in the Physical Environment	“Having a yoga mat at home is also more motivation since it sits in right by my door and I pass it every day.”
Sustenance	Emotional Transformation	“The one main thing that yoga helped me realize is that the stress will still be there no matter how much I think about it and that it’s always better to do yoga when you are stressed because you need to give yourself time to be less tense.” (directing negative emotions into purposes)
	Practice for Change	“I recently just signed up at (studio/gym) and have been attending their yoga (sustained commitment to the practice) and I love it so much. Yoga has really helped me make many good changes in my life.”
	Changes in the Social Environment	“I thought the instructor was very helpful and understanding (social support) when it came to people’s abilities as beginners in yoga.”

## Data Availability

The data presented in this study are available upon request from the corresponding author. The data are not publicly available due to the presence of ethical reasons.
